# Superconducting Properties in Arrays of Nanostructured *β*-Gallium

**DOI:** 10.1038/s41598-017-15738-2

**Published:** 2017-11-10

**Authors:** K. O. Moura, K. R. Pirota, F. Béron, C. B. R. Jesus, P. F. S. Rosa, D. Tobia, P. G. Pagliuso, O. F. de Lima

**Affiliations:** 1 Instituto de Física Gleb Wataghin, UNICAMP, Campinas, SP 13083-859 Brazil; 2 Programa de Pós-Graduação em Física, Campus Prof. José Aluísio de Campos, UFS, 49100-000, São Cristóvão, SE Brazil; 3 Condensed Matter and Magnet Science, Los Alamos National Laboratory, Los Alamos, New Mexico 87545 USA

## Abstract

Samples of nanostructured *β*-Ga wires were synthesized by a novel method of metallic-flux nanonucleation. Several superconducting properties were observed, revealing the stabilization of a weak-coupling type-II-like superconductor (${T}_{c}$ $\approx$ 6.2 K) with a Ginzburg-Landau parameter ${\kappa }_{GL}$ = 1.18. This contrasts the type-I superconductivity observed for the majority of Ga phases, including small spheres of *β*-Ga with diameters near 15 μm. Remarkably, our magnetization curves reveal a crossover field ${H}_{D}$, where we propose that the Abrikosov vortices are exactly touching their neighbors inside the Ga nanowires. A phenomenological model is proposed to explain this result by assuming that only a single row of vortices is allowed inside a nanowire under perpendicular applied field, with an appreciable depletion of Cooper pair density at the nanowire edges. These results are expected to shed light on the growing area of superconductivity in nanostructured materials.

## Introduction

Pure bulk gallium, usually known as *α*-Ga, has a stable orthorhombic structure at room temperature and is a type-I superconductor with critical temperature (${T}_{c}$) around 1.08 K^1,2^. Elemental Ga, however, presents a large degree of polymorphism with more than ten different crystalline phases^2–4^ which are dependent on temperature, pressure and geometrical confinement. The majority of Ga phases exhibits type-I superconductivity. An interesting example is *β*-Ga that shows ${T}_{c}$ $\approx$ 6.2 K, observed^5,6^ for small spheres with diameters around 15 $\mu$m. The *β*-Ga phase is metastable at atmospheric pressure and has a monoclinic structure with melting temperature of 256.8 K^7,8^.

About 50 years ago, theoretical^9^ and experimental^10^ studies found that a type-II-like behavior should exist when a perpendicular magnetic field is applied to sufficiently thin films of any superconducting material. Indeed, it was experimentally verified that thin films of type I materials, such as Pb, Sn and In present type-II behavior for thicknesses below 250 nm, 180 nm and 80 nm, respectively^4,10^. More recently, with the help of advanced instrumentation, special techniques and powerful computer simulations, unexpected new results in this area have been reported in studies done on mesoscopic samples^11–17^. The relevance of sample topology on the nucleation of superconductivity was clearly demonstrated in mesoscopic aluminum samples^11^ and a variety of vortex patterns were observed and calculated using the linearized Ginzburg-Landau (GL) equations, with appropriate boundary conditions^12^. Geometry-driven vortex states were also observed in lead nanowires^13^, as well as in micron-size *β*-Sn samples^14^, and interpreted with three-dimensional GL simulations.

Here we report results on a nanostructured array of *β*-Ga synthesized by a novel method of metallic-flux nanonucleation (MFNN)^18–20^. The superconducting properties measured in our samples are interpreted taking into account a type-II-like behavior. Some features derived from the Abrikosov vortices system, are also discussed.

## Results

**Figure 1:**
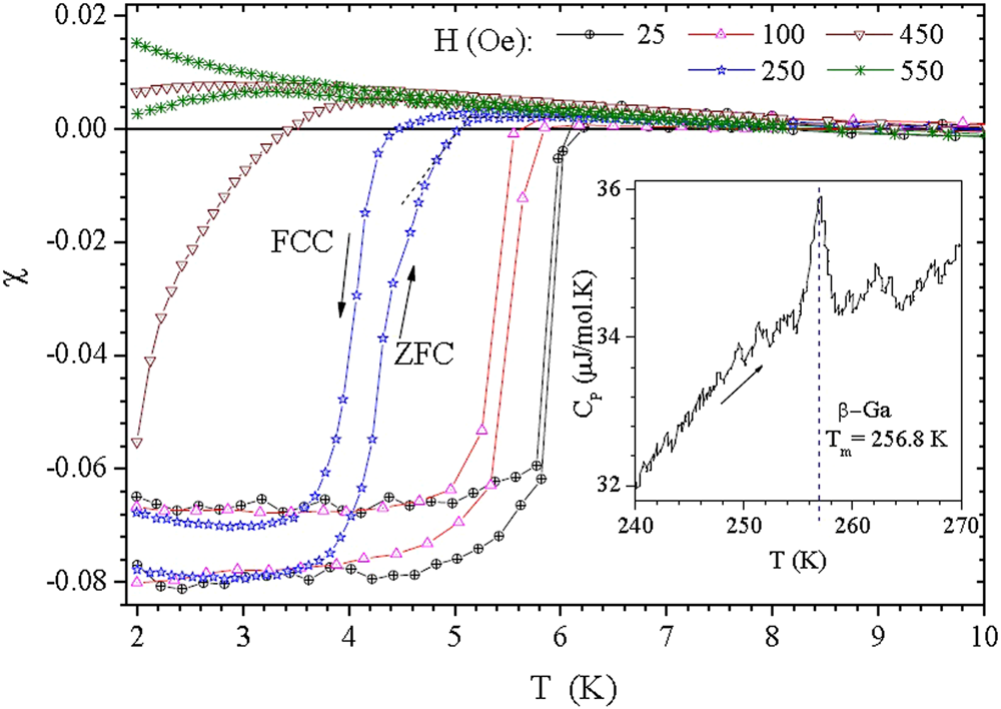
Magnetic susceptibility as a function of temperature for the nGa sample. The onset of transition is defined at the intersection of the zero field cooling (ZFC) curve and the normal paramagnetic line, as illustrated by the dashed straight lines on the 250 Oe curve. Inset: Specific heat at constant pressure measured on warming. The peak indicates the latent heat at the melting point of *β*-Ga.

Magnetization curves, as a function of temperature (*MT*) and magnetic field (*MH*), were measured in a Quantum Design SQUID (Superconducting Quantum Interference Device) and PPMS, respectively. Specific heat was measured using a two-relaxation-times technique in the PPMS. All magnetization measurements shown here were taken with *H* perpendicular to the nanowire array. Figure 1 displays some of the *MT* converted to susceptibility ($\chi$ = *M/H*) and normalized to $-\mathrm{1/4}\pi$ at the saturated maximum shielding of zero field cooling (ZFC) measurements, for the lowest applied fields. The paramagnetic background in the normal state region was not subtracted since it does not interfere with the analysis.

The sharp transitions at low fields that define a ${T}_{c}$ of 6.2 K (Fig. 1) and the nanowire diameter of 140 nm (within the range of sizes^21,22^ that favors the stabilization of *β*-Ga) are indications that we obtained a pure *β*-Ga phase. Perhaps most importantly, the graph shown in the inset of Fig. 1 displays the specific heat of the nGa sample, measured on warming. The peak at *T*_*m*_ = 256.8 K represents the latent heat associated^22^ with melting of the pure *β*-Ga phase.

**Figure 2:**
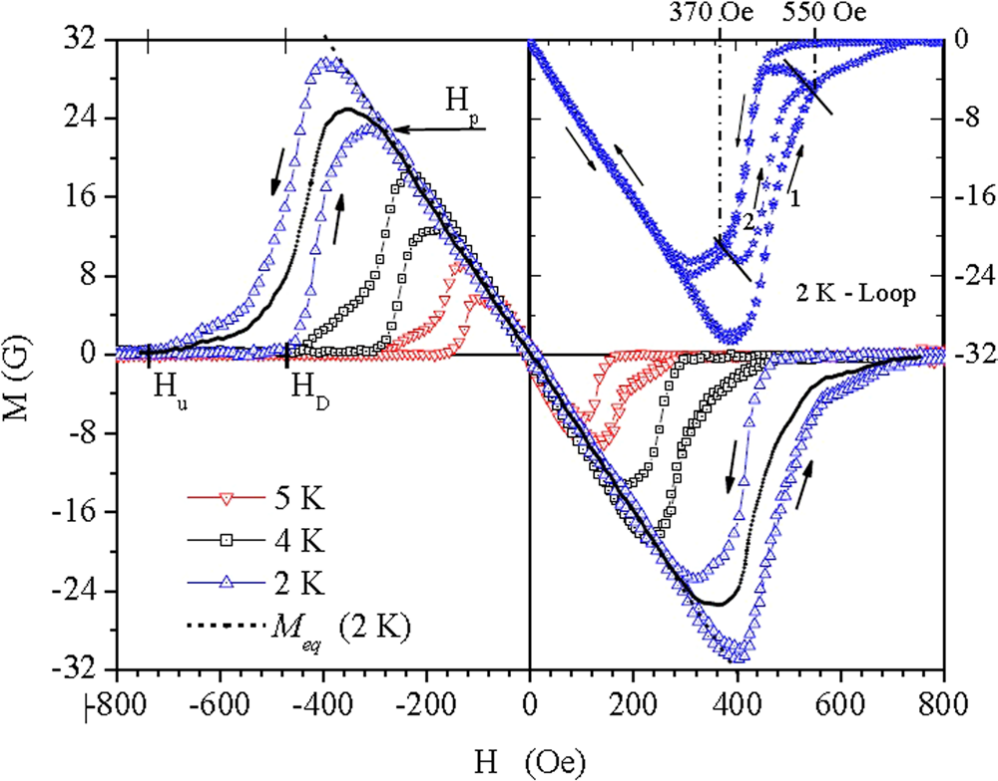
Magnetization as a function of perpendicular applied field. Definitions for the penetration field (*H*_*p*_), upper critical field (*H*_*u*_) and crossover field (*H*_*D*_) are shown. The curve for 2 K is repeated (open stars) between 0 and 800 Oe to execute a minor hysteresis loop (see text).

Figure 2 displays a set of *MH* curves for *T* = 2 K, 4 K and 5 K. These curves were obtained by subtracting the normal paramagnetic background coming from the alumina template and nanowires. First, a normal state reference curve, obtained for the nGa sample at 6.2 K, was subtracted from each curve measured in the superconducting state for the same sample. In this process the signal magnitude was properly corrected to account for the temperature dependence. Second, we performed an additional subtraction of the paramagnetic contribution coming from the unfilled alumina template, which was measured at each *T* of interest. Therefore, the final *MH* curves shown in Fig. 2 are attributed solely to the Ga nanowires.

The *MH* curves show a large hysteresis between the ascending (M $\uparrow$) and descending (M $\downarrow$) branches, as indicated by the arrows near the 2 K curve. Equilibrium magnetization curves can be evaluated by the average^23,24^ ${M}_{eq}$ = (M $\uparrow$ + M $\downarrow$)/2. One calculated example (at 2 K) is plotted as a black dashed line in Fig. 2. A penetration field *H*_*p*_ is defined at the point where *M*_*eq*_ departs from the Meissner state straight line, and an upper critical field *H*_*u*_ is defined at the merging point with the normal state baseline.

A relevant fact is that only a very small hysteresis appears between the *MH* curves for the first increase from *H* = 0 (virgin state) and subsequent field increases. This could be due to a negligible bulk pinning of vortices as they enter the nanowires in a similar way^25^, independently of the field cycling. Under decreasing field, however, a practically zero magnetization is observed, as expected from the Bean-Livingston (BL) surface barrier mechanism^24,26^, until a crossover value *H*_*D*_ is reached and diamagnetic shielding currents show up. This strong asymmetry, between the ascending and descending branches of *MH* curves, indicates^24^ the dominance of the BL barrier over the negligible bulk pinning. It is important to mention that *MH* curves measured with *H* parallel to the nanowires (not presented here) do not show a crossover field like *H*_*D*_.

To further explore the magnetization behavior of the nGa samples a minor hysteresis loop^27,28^, was measured on top of the second *MH* curve at $T=2K$, represented by open stars in the first quadrant of Fig. 2, with an inverted vertical scale at the right axis. This curve starts at *H* = 0 going up to 550 Oe (arrow 1), then is reversed down to 370 Oe, then reversed up to 550 Oe (arrow 2) and then reversed down to *H* = 0. This completes the full loop, which almost overlaps with the first measured *MH* curve represented by open up-triangles. The relevant feature in the whole process is the minor hysteresis loop between 550 Oe and 370 Oe, showing that a substantial portion of the reversed branches (down and up) are almost parallel to the Meissner straight line. These portions are marked in the graph by two straight line segments that indicate the dominance of the surface barrier against the entrance of vortices^29^.

**Figure 3:**
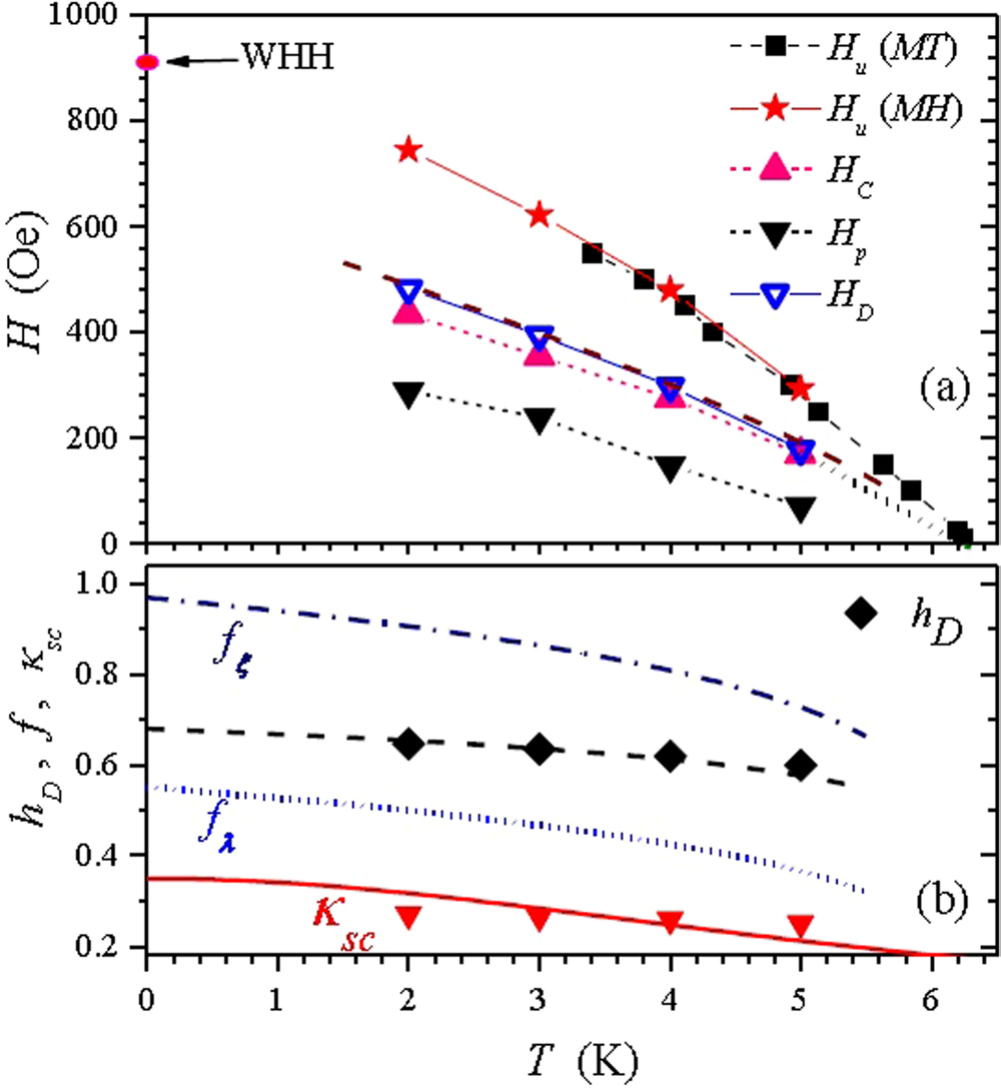
(**a**) Field lines for *H*_*p*_, *H*_*u*_, *H*_*c*_ and *H*_*D*_. The dashed and straight segments joining the points are only guides to the eyes. (**b**) Closed black diamonds represent the reduced crossover field ${h}_{D}={H}_{D}/{H}_{u}$ and the dashed line represent the fitted function for ${h}_{D}$. The dash-dotted and dotted lines represent the calculated depletion parameters ${f}_{\xi }$ and ${f}_{\lambda }$, respectively (see text). Solid down triangles represent the supercooling kappa parameter ${\kappa }_{sc}$ and the solid line is a two fluid model fit through these points.

Figure 3(a) presents plots for the fields *H*_*p*_, *H*_*u*_, and *H*_*D*_ whose data (see Table 1) were extracted from Figs 1 and 2 within an experimental error of 5%. Notice the good agreement between *H*_*u*_ lines extracted from *MH* curves (closed stars) and *MT* curves (closed squares).

**Table 1:** Superconducting parameters extracted from Figs 1 and 2.

*T*	*H*_*p*_	*H*_*u*_	*H*_*c*_	*H*_*D*_	*h*_*D*_
(K)	(Oe)	(Oe)	(Oe)	(Oe)	
2	288	745	438	482	0.647
3	239	622	355	395	0.635
4	148	480	275	298	0.621
5	71	293	169	178	0.607

## Discussion

We present in this section different possible interpretations for our data. It is important to notice that our magnetization curves represent a global response of the total nanowire array. Due to the high uniformity of the nGa sample, however, we infer that all properties calculated in this section are the same for each individual nanowire, which are separated by the insulating matrix. Also, because the quantized flux lines cross each nanowire along its length and have comparable diameter sizes, there will be a depletion of Cooper pair density^30^ at the wire edges. This produces effective values for the coherence length ${\xi }_{e}(T)={f}_{\xi }(T)\xi (T)$ and penetration depth ${\lambda }_{e}(T)={f}_{\lambda }(T)\lambda (T)$. Here, $\xi (T)$ and $\lambda (T)$ are the usual 3D parameters from Ginzburg-Landau (GL) theory and ${f}_{\xi }(T)$, ${f}_{\lambda }(T)$ are depletion parameters to be determined from the experimental data.

Assuming that *H*_*u*_ is similar to the bulk nucleation field *H*_*c2*_ from GL theory, we estimate *H*_*u*_(0) = 923 Oe, at $T=0$, from the WHH formula^31^ *H*_*c2*_(0) = −0.693 *T*_*c*_(d *H*_*u*_/dT)_*Tc*_, where the slope of *H*_*u*_ at *T*_*c*_ is −215 Oe/K. Also, we adopt the GL expression for the upper critical field ${H}_{u}(T)={{\rm{\Phi }}}_{0}/[2\,\pi {\xi }_{e}^{2}(T)]$, where ${{\rm{\Phi }}}_{0}$ = $2.07\times {10}^{-7}$ $Gc{m}^{2}$ is the flux quantum. Then, the effective coherence length at *T* = 0 becomes ${\xi }_{e}\mathrm{(0)}$ $\approx$ 60 nm. This means that a vortex core at T = 0 has a diameter just slightly smaller than that of the Ga nanowire.

Table 1 and Fig. 3 also show values for the thermodynamic critical field (${H}_{c}$), calculated with an equation that balances the isothermal magnetic work and the condensation energy involved in the superconducting transition^32^:

1 $${\int }_{0}^{{H}_{u}}{M}_{eq}dH=-\frac{{H}_{c}^{2}}{8\pi }$$

With ${H}_{c}(T)$, we can then estimate the Maki parameter ${\kappa }_{1}(T)={H}_{u}(T)/[\sqrt{2}{H}_{c}(T)]$, which was introduced in a pioneering work^33^ to extend the GL theory to $0 < T < {T}_{c}$. Some estimated values of ${\kappa }_{1}(T)$ are 1.24 (3 K), 1.23 (4 K) and 1.21 (5 K). This decreasing trend when T increases is in fact the expected trend^34^. Extrapolating to ${T}_{c}$ we obtain the value ${\kappa }_{1}({T}_{c})={\kappa }_{GL}$ = 1.18, where ${\kappa }_{GL}$ is the original Ginzburg Landau parameter.

The equation^35^ ${H}_{u}\mathrm{(0)}\approx 1.77{\kappa }_{GL}{H}_{c}\mathrm{(0)}$ relates the upper critical field and thermodynamical critical field, both at T = 0 with the GL parameter at ${T}_{c}$ and yields ${H}_{c}\mathrm{(0)}$ ≈ 442 Oe. We can then get the effective penetration depth from^32^ ${\lambda }_{e}\mathrm{(0)}={[{{\rm{\Phi }}}_{0}{H}_{u}\mathrm{(0)/4}\pi {H}_{c}^{2}\mathrm{(0)]}}^{0.5}$ ≈ 88 nm.

The ratio between the energy gap for a Cooper pair at $T=0$ and the thermal energy at ${T}_{c}$, which is an important parameter from BCS theory, can now be calculated by^36^:

2 $$\frac{2{\rm{\Delta }}\mathrm{(0)}}{k{T}_{c}}=-\frac{2{T}_{c}}{{H}_{c}\mathrm{(0)}}{(\frac{d{H}_{c}}{dT})}_{{T}_{c}}\approx 3.61$$

where the slope of ${H}_{c}(T)$ at ${T}_{c}$ was evaluated (Fig. 3) to be around −129 Oe/K. This value of 3.61 for *β*-Ga is close to the BCS prediction of 3.53 for weak-coupling superconductors^32,37^, and is similar to In (3.63), Sn (3.6) and Ta (3.6).

*A Model for H*_*D*_. - From the calculated properties above, we conclude that our nGa sample is consistently well described as a weak-coupling type-II-like superconductor. The estimated values of ${\xi }_{e}\mathrm{(0)}\approx 60$ nm and ${\lambda }_{e}\mathrm{(0)}\approx 88$ nm suggest that at very low *T* the diameter of the vortices nearly matches the nanowire diameter of 140 nm. When temperature increases ${\xi }_{e}(T)$ and ${\lambda }_{e}(T)$ become increasingly depleted^30^. This effect is especially pronounced along the nanowire diameter, because there is no severe size restrictions along the nanowire length. This leads to the conclusion that only one row of vortices is allowed inside the nanowire. This is similar to the reported scenario for Pb nanowires of diameters near 390 nm, under perpendicular *H* ^13^. For thicker Pb nanowires^13^ or millimeter-sized disks^17^ a classical type-I intermediate state with multiquanta domains are observed.

**Figure 4:**
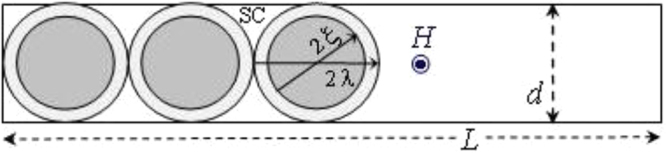
Model of vortices row crossing a nanowire with the field *H* applied perpendicularly to its length (see text).

We propose a simple phenomenological model assuming that the crossover field *H*_*D*_ corresponds to the situation in which the vortices are exactly touching their neighbors as depicted in Fig. 4. Because this happens in the descending branch of *MH* curves, it is helpful to recall that overlapped vortices are nucleated at *H*_*u*_ and become gradually separated as *H* decreases. This occurs because part of the vortices leaves the nanowire easily, with no surface barrier, as discussed before. When $H\le {H}_{D}$, the superconducting regions are enhanced between the vortices, producing a fast increase of the diamagnetic response as observed (see Fig. 2).

In Fig. 4, when *H*_*D*_ is reached, a geometrical relation between the nanowire length (*L*) and the number of enclosed vortices inside (*N*_*v*_) will be $L={N}_{\nu }2\lambda$. The density of vortices will be $n={N}_{\nu }/Ld$, using the maximal longitudinal cross-section area of the nanowire. Since ${H}_{D}=B=n{{\rm{\Phi }}}_{0}$, where *B* is the magnetic induction, we get ${H}_{D}={{\rm{\Phi }}}_{0}/\mathrm{(2}d\lambda )$. Employing^32^ $\lambda (t)=\lambda \mathrm{(0)[2(1}-t{)]}^{-0.5}$, with $t=T/{T}_{c}$, and expressing the penetration depth in terms of the effective (experimental) ${\lambda }_{e}(t)$ we get:

3 $${H}_{D}(t)=\frac{{f}_{\lambda }(t)\sqrt{2}{{\rm{\Phi }}}_{0}}{2d{\lambda }_{e}\mathrm{(0)}}{\mathrm{(1}-t)}^{0.5}$$

A fit to the experimental data gives ${H}_{D}(t)=\mathrm{655(1}-t{)}^{0.75}$, represented by the dashed line in Fig. 3(a). Combining this result with equation (3) we get ${f}_{\lambda }(t)=\mathrm{0.551(1}-t{)}^{0.25}$, which is plotted in Fig. 3(b) as a dotted line.

The ratio ${h}_{D}={H}_{D}/{H}_{u}$ is an interesting parameter that can be used to test our model. Using equation (3), ${f}_{\lambda }(t)$ and ${H}_{u}(t)$ we get:

4 $${h}_{D}(t)=\frac{{f}_{\lambda }(t){f}_{\xi }^{2}(t)\sqrt{2}\pi {\xi }_{e}^{2}\mathrm{(0)}}{d{\lambda }_{e}\mathrm{(0)}}{\mathrm{(1}-t)}^{-0.5}$$

As shown in Table 1 and Fig. 3(b) ${h}_{D}$ decreases just slightly and almost linearly as *T* increases in the measured range. A fit to the experimental data gives ${h}_{D}=\mathrm{0.68(1}-t{)}^{0.1}$, represented by a dashed line in Fig. 3(b). Combining this result with equation (4) we get ${f}_{\xi }(t)=\mathrm{0.975(1}-t{)}^{0.175}$, which is plotted in Fig. 3(b) as a dot-dashed line.

The weakly decreasing monotonic behavior of ${f}_{\xi }$ and ${f}_{\lambda }$, as *T* increases in the measured range, is in fact inversely proportional to the depletion degree of Cooper pair density in the vortex volume. This is the expected trend. As *T* grows both $\lambda (T)$ and $\xi (T)$ must increase, eventually exceeding the nanowire diameter. This causes an enhanced depletion at the edges^30^.

We have also tried to interpret our data as type-I superconductivity, similar to the approach used in ref.^6^ for $\beta  \mbox{-} Ga$ microspheres; by assuming the upper critical field (${H}_{u}$) to be ${H}_{cI}$ and the crossover field (*H*_*D*_) to be a supercooling field (${H}_{sc}$). Because^6,15^ ${H}_{sc}$ $\approx$ 2.39 ${\kappa }_{sc}{H}_{cI}$ the supercooling kappa parameter can be calculated by ${\kappa }_{sc}\approx {h}_{D}\mathrm{/2.39}$, which is plotted as solid down triangles in Fig. 3(b). The solid line through these points is a fit of the expected two fluid model expression^14,15^ ${\kappa }_{sc}(t)\approx {\kappa }_{sc}\mathrm{(0)}/\mathrm{(1}+{t}^{2})$, where ${\kappa }_{sc}\mathrm{(0)}$ = 0.35. Clearly this temperature dependence does not fit well our data. Also, one can obtain ${H}_{cI}\mathrm{(0)}$ ≈ 820 Oe by fitting a parabolic expression to our ${H}_{u}$ data in Fig. 3(a). Using the GL expression^25^ ${H}_{c}={{\rm{\Phi }}}_{0}/\mathrm{(2}\pi \sqrt{2}\lambda \xi )$ and $\xi =\lambda /{\kappa }_{sc}$ one gets $\lambda \mathrm{(0)}\approx 315$ nm and $\xi \mathrm{(0)}\approx 90$ nm. These results sound unlikely and are very different from those found in ref.^6^ for $\beta  \mbox{-} Ga$ microspheres. Particularly, in the type-I interpretation, the BCS energy ratio would be 2 ${\rm{\Delta }}\mathrm{(0)}/k{T}_{c}\,\approx$ 6, because the slope ${(d{H}_{cI}/dT)}_{Tc}$ ≈ −215 Oe/K has to be used instead of ${(d{H}_{c}/dT)}_{Tc}$ ≈ −129 Oe/K in equation (2). However this energy ratio value is unrealistically high, even for a strong coupling superconductor.

## Conclusion

Samples of nanostructured *β*-Ga wires were successfully prepared by a novel method of metallic-flux nanonucleation. Several superconducting properties were determined from magnetization measurements and are well described as a weak-coupling type-II-like superconductor with a Ginzburg-Landau parameter ${\kappa }_{GL}$ = 1.18.

Possibly the unexpected type-II-like behavior reported here is favored by the nanoscopic scale of the Ga nanowires, stabilized in very particular geometrical conditions. To our knowledge, no such effect has yet been verified for Ga. Particularly we have introduced a model to interpret a clearly defined crossover field (*H*_*D*_), using simple ideas based on the GL theory and vortex behavior. Although the obtained results seems plausible, we feel that a more accurate and fundamental treatment is lacking, especially to explain the depletion parameters ${f}_{\xi }$ and ${f}_{\lambda }$, introduced to take account of the partial suppression of the vortex volume (or Cooper pair density^30^) at the nanowire edges.

We also tried to interpret the data as a classical type-I superconductor, but the results were not so convincing. We then conclude that possibly our $\beta  \mbox{-} Ga$ nanowires, under perpendicular applied field, favors a type-II-like behavior that calls for further investigation. We are planning to study new $\beta  \mbox{-} Ga$ samples with different nanowires diameters, as well as samples of Sn and In, prepared by the same method employed here. Finally, we hope this work will motivate new studies regarding nanostructured superconductors^38^.

## Methods

**Figure 5:**
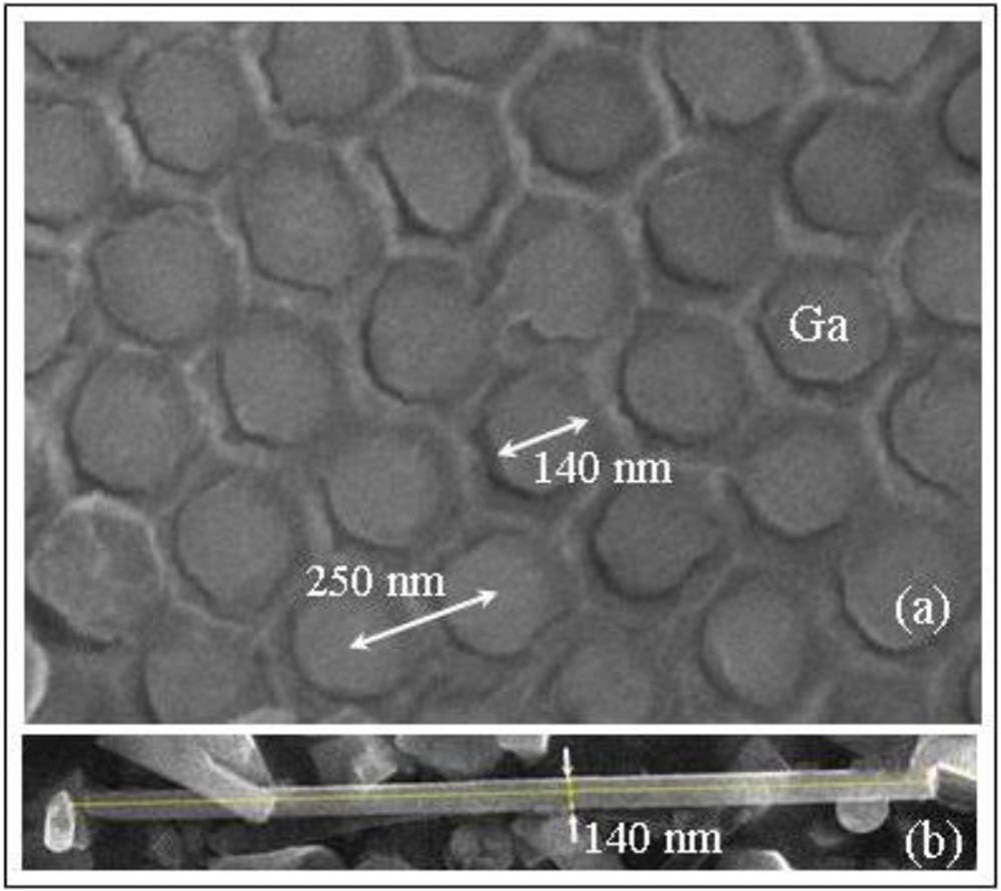
SEM images of (**a**) a small area of the nGa sample showing the triangular array of Ga nanowires in top view and (**b**) a longitudinal view of one Ga nanowire.

The MFNN technique^18–20^ has been successfully developed to nucleate crystalline nanowires inside the pores of an alumina template. The nanoporous template presents several advantages, such as an excellent pore-size control over large areas and large aspect-ratio pores that exhibit a highly regular spatial pattern. Our present samples consist of small pieces of the alumina template filled with pure Ga (nGa), having typically an area of 2 by 2 mm^2^ and thickness of 80 $\mu$m. Figure 5(a),(b), show Scanning Electron Microscope (SEM) images of a small top view area and a longitudinal view of one Ga nanowire, respectively. The nanowires were exposed by gently crushing a filled template. Figure 5(b) shows a nanowire with uniform diameter of 140 nm and length around 3.8 $\mu$m. This is only a small portion from one of the original wires embedded in the template, which are typically 80 $\mu$m long. The distances between the centers of the neighboring nanowires are fixed at 250 nm, forming a nearly perfect triangular array as shown in Fig. 5(a).

## References

[1] de Haas WJ, Voogd J (1929). On the superconductivity of the gallium. Commun. Phys. Lab. Univ. Leiden.

[2] Roberts BW (1976). Survey of superconductive materials and critical evaluation of selected properties. J. Phys. Chem. Ref. Data.

[3] Teske D, Drumheller JE (1999). Phases of gallium nucleated by small particles. J. Phys.: Condens. Matter.

[4] Charnaya EV, Tien C, Lee MK, Kumzerov YA (2009). Superconductivity and structure of gallium under nanoconfinement. J. Phys.: Condens. Matter.

[5] Feder J, Kiser SR, Rothwarf F, Burger JP, Valette C (1966). Hysteresis effects in three superconducting phases of gallium. Solid State Commun..

[6] Parr H, Feder J (1973). Superconductivity in β-phase gallium. Phys. Rev. B.

[7] Bosio L, Defrain A, Epelboin I (1966). Changements de phase du gallium a la pression atmosphérique. J. Phys. (Paris).

[8] Bosio L, Defrain A (1969). Structure cristalline du gallium *β* Acta Crystallogr. Sect. B.

[9] Tinkham M (1963). Effect of fluxoid quantization on transitions of superconducting films. Phys. Rev..

[10] Dolan GJ, Silcox J (1973). Critical thicknesses in superconducting thin films. Phys. Rev. Lett..

[11] Moshchalkov VV (1995). Effect of sample topology on the critical fields of mesoscopic superconductors. Nature.

[12] Chibotaru LF (2005). Ginzburg-Landau description of confinement and quantization effects in mesoscopic superconductors. J. Math. Phys..

[13] Engbarth MA, Bending SJ, Milosevic MV (2011). Geometry-driven vortex states in type-I superconducting Pb nanowires. Phys. Rev. B.

[14] Müller A, Milosevic MV, Dale SEC, Engbarth MA, Bending SJ (2012). Magnetization measurements and Ginzburg-Landau simulations of micron-size *β*-tin samples: evidence for an unusual critical behavior of mesoscopic type-I superconductors. Phys. Rev. Lett..

[15] Lukyanchuk I (2015). Rayleigh instability of confined vortex droplets in critical superconductors. Nature Physics.

[16] Roditchev D (2015). Direct observation of Josephson vortex cores. Nature Physics.

[17] Prozorov R (2007). Equilibrium topology of the intermediate state in type-I superconductors of different shapes. Phys. Rev. Lett..

[18] Pirota, K. R. *et al*. *Processo de produção de nanofios monocristalinos intermetálicos*. BR patent 10 2014 019794 0 issued 11 Aug. 2014 (international patent pending WO2016023089 A1).

[19] Rosa PFS (2014). Exploring the effects of dimensionality on the magnetic properties of intermetallic nanowires. Solid State Commun..

[20] Moura KO (2016). Dimensionality tuning of the electronic structure in *Fe*_3_*Ga*_4_ magnetic materials. Scientific Reports.

[21] Li XF (2011). Size-temperature phase diagram of gallium. Europhys. Lett..

[22] Di Cicco A, Fusari S, Stizza S (1999). Phase transitions and undercooling in confined gallium. Philos. Mag. B.

[23] Chaddah P, Roy SB, Chandran M (1999). Inferring equilibrium magnetization from hysteretic *M-H* curves of type-II superconductors. Phys. Rev. B.

[24] Konczykowski M, Burlachkov I, Yeshurun Y, Holtzberg F (1991). Evidence for surface barriers and their effect on irreversibility and lower-critical-field measurements in Y-Ba-Cu-O crystals. Phys. Rev. B.

[25] Blatter G, Feigeľman MV, Geshkenbein VB, Larkin AI, Vinokur VM (1994). Vortices in high-temperature superconductors. Rev. Mod. Phys..

[26] Bean CP, Livingston JD (1964). Surface barrier in type-II superconductors. Phys. Rev. Lett..

[27] Freyhardt HC, Haasen P (1967). Magnetische flussgradienten in verformten niob-einkristallen bei 4.2 K. Z. Metallkd..

[28] Brito AS, Zerweck G, de Lima OF (1979). High critical flux density gradients near the surface of superconducting niobium. J. Low Temp. Phys..

[29] Ullmaier, H. In *Irreversible Properties of Type II Superconductors* 123-125 (Springer-Verlag, Berlin, Heidelberg, 1975).

[30] Romaguera ARC, Doria MM, Peeters FM (2007). Tilted vortices in a superconducting mesoscopic cylinder. Phys. Rev. B.

[31] Werthamer NR, Helfand E, Hohenberg PC (1966). Temperature and purity dependence of the superconducting critical field,*H*_c2_. III. Electron spin and spin-orbit effects. Phys. Rev..

[32] Tinkham M. pp. 63, 120, 135, 161 in *Introduction to Superconductivity* (McGraw-Hill, New York, 1996).

[33] Maki K (1964). Magnetic properties of superconducting alloys. I. Physics.

[34] Fetter, A. L. & Hohenberg, P. C. *In: Superconductivity*, v. 2, p. 817, ed. R. D. Parks (Marcel Dekker, Inc., New York, 1969).

[35] Goŕkov LP (1960). The critical supercooling field in superconductivity theory. Sov. Phys. JETP.

[36] Toxen AM (1965). New relationship between the critical field and energy gap of a superconductor. Phys. Rev. Letters.

[37] Lynton, E. A. The energy gap In *Superconductivity* 95–115 (Methuen Monograph, London, 1969).

[38] Moshchalkov, V. V. & Fritzsche, J. *Introduction. In Nanostructured Superconductors* 3–11 (World Scientific Publishing Co., Singapore 2011).

